# Methyltransferase-like 3 aggravates endoplasmic reticulum stress in preeclampsia by targeting TMBIM6 in YTHDF2-dependent manner

**DOI:** 10.1186/s10020-023-00604-x

**Published:** 2023-02-06

**Authors:** Yangyang Chen, Xiaoxia Liu, Lun Li, Xiyang He, Fanghui Zheng, Yang Zhang, Hui Gao, Zhishan Jin, Di Wu, Qianhua Wang, Hui Tao, Yin Zhao, Weifang Liu, Li Zou

**Affiliations:** Department of Obstetrics and Gynecology, Union Hospital, Tongji Medical College, Huazhong University of Science and Technology, Wuhan, 430022 China

**Keywords:** Preeclampsia, m^6^A methylation, Endoplasmic reticulum stress, METTL3, YTHDF2, TMBIM6

## Abstract

**Background:**

With the increasing morbidity and mortality of preeclampsia (PE), it has posed a huge challenge to public health. Previous studies have reported endoplasmic reticulum (ER) stress could contribute to trophoblastic dysfunction which was associated with the N^6^-methyladenosine (m^6^A) modification by methyltransferase-like 3 (METTL3), resulting in PE. However, little was known about the relationship between METTL3 and ER stress in PE. Thus, in vitro and in vivo studies were performed to clarify the mechanism about how METTL3 affects the trophoblasts under ER stress in PE and to explore a therapeutic approach for PE.

**Methods:**

An ER stress model in HTR-8/SVneo cells and a preeclamptic rat model were used to study the mechanism and explore a therapeutic approach for PE. Western blot, immunohistochemistry, quantitative reverse transcription-polymerase chain reaction (qRT-PCR), and methylated RNA immunoprecipitation (MeRIP)-qPCR were performed to detect the protein, RNA, and methylated transmembrane BAX inhibitor motif containing 6 (TMBIM6) expression levels. The m^6^A colorimetric and mRNA stability assays were used to measure the m^6^A levels and TMBIM6 stability, respectively. Short hairpin RNAs (shRNAs) were used to knockdown METTL3 and YTH N6-methyladenosine RNA binding protein 2 (YTHDF2). Flow cytometry and Transwell assays were performed to evaluate the apoptosis and invasion abilities of trophoblasts.

**Results:**

Upregulated METTL3 and m^6^A levels and downregulated TMBIM6 levels were observed in preeclamptic placentas under ER stress. The ER stress model was successfully constructed, and knockdown of METTL3 had a beneficial effect on HTR-8/SVneo cells under ER stress as it decreased the levels of methylated TMBIM6 mRNA. Moreover, overexpression of TMBIM6 was beneficial to HTR-8/SVneo cells under ER stress as it could neutralize the harmful effects of METTL3 overexpression. Similar to the knockdown of METTL3, downregulation of YTHDF2 expression resulted in the increased expression and mRNA stability of TMBIM6. Finally, improved systemic symptoms as well as protected placentas and fetuses were demonstrated in vivo.

**Conclusions:**

METTL3/YTHDF2/TMBIM6 axis exerts a significant role in trophoblast dysfunction resulting in PE while inhibiting METTL3 may provide a novel therapeutic approach for PE.

**Supplementary Information:**

The online version contains supplementary material available at 10.1186/s10020-023-00604-x.

## Background

Preeclampsia (PE) is a devastating pregnancy-specific disorder characterized by new-onset hypertension and multisystem end-organ damage, often accompanied by proteinuria after the 20th week of gestation (Ives et al. 2020). PE affects 2–8% of pregnant women globally and is associated with serious maternal and neonatal morbidity and mortality (Abalos et al. 2014). Although PE is a pregnancy-specific syndrome, its pathogenesis is not yet completely understood.

Inadequate trophoblast invasion and deficient remodeling of spiral arteries can lead to placental ischemia–reperfusion (I/R) injury, which in turn promotes the pathogenesis of PE (Rana et al. 2019; Zhao et al. 2021). Placental I/R injury can induce trophoblast apoptosis by increasing the accumulation of unfolded or misfolded proteins in the endoplasmic reticulum (ER), triggering ER stress (Bastida-Ruiz et al. 2017). In response to ER stresm, cells activate a series of signaling pathways, known as the unfolded protein response (UPR), to maintain the structural and functional homeostasis of ER. However, if ER stress is sustained and the UPR fails to eliminate the unfolded/misfolded proteins, stressed cells undergo self-destruction, which may be a key cause of various diseases, including inflammatory diseases, neurodegenerative diseases, metabolic diseases, and cancer (Wang and Kaufman 2016). Several studies have proved the relationship between excessive ER stress and PE as well as the levels of various ER stress-associated proteins, including activating transcription factor (ATF)-6, ATF4, C/EBP homologous protein (CHOP), protein kinase R (PKR)-like ER kinase (PERK), and glucose-regulated protein 78 (GRP78), increased in the placenta during PE (Bastida-Ruiz et al. 2017; Kawakami et al. 2014). However, the specific molecular and cellular mechanisms linking ER stress to PE are not yet completely understood.

The transmembrane Bax inhibitor motif containing 6 (TMBIM6), also known as Bax interactivator-1 (BI-1), is a highly conserved transmembrane protein primarily located in the ER (Lebeaupin et al. 2020). TMBIM6 inhibits Bax-induced apoptosis, shows beneficial effects against ER stress and calcium imbalance, and possesses anti-inflammatory and anti-oxidative properties (Lebeaupin et al. 2020; Han et al. 2021; Bhattarai et al. 2021). TMBIM6 can inhibit ER stress-induced apoptosis via multiple mechanisms, such as by regulating the calcium flux in the ER lumen, activating the PERK/ATF4/CHOP pathway, and limiting reactive oxygen species (ROS) production (Lebeaupin et al. 2020). Several studies have investigated the roles of TMBIM6 in various diseases, including diabetes, severe acute respiratory syndrome-coronavirus-2 infection, Alzheimer’s disease, and cancer (Han et al. 2021; Wang et al. 2021a; Yadav et al. 2015; Chang et al. 2021; Nho et al. 2020; Zhou et al. 2020). However, whether TMBIM6 is associated with the occurrence and development of PE remains unknown.

N6-methyladenosine (m^6^A) is one of the most prevalent methylation modifications in mRNA that is involved in moderating the RNA structure, function, metabolism, and stability, thereby affecting the expression of many genes. Three types of proteins composed of “writers,” “erasers,” and “readers” are involved in the methylation modification process affecting the m^6^A levels of RNA and associated with various diseases, including cancer, cardiovascular diseases, I/R injury, and diabetes (Oerum et al. 2021; Suo et al. 2022; Chien et al. 2021; Yao et al. 2020; Qin et al. 2022). However, whether the m^6^A modification has beneficial or harmful effects on ER stress-associated diseases remains unclear. Some studies have suggested that the WT1-associated protein-mediated m^6^A modification of ATF4 mRNA exacerbates myocardial I/R injury by increasing the ER stress and cell apoptosis. In addition, methyltransferase-like (METTL)-14 can facilitate liver regeneration by decaying CHOP mRNA via its m^6^A modification (Wang et al. 2021b; Cao et al. 2021). However, little is known about the effects of RNA modifications on trophoblast function under ER stress in PE.

METTL3 is the best known m^6^A methyltransferase that plays an important role in the reversible epigenetic regulation of the m^6^A modification (Liu et al. 2020). Several sequencing and mechanistic studies have shown that METTL3 levels are upregulated in PE, and it aggravates disease progression by affecting the trophoblast proliferation, migration, and invasion (Wang et al. 2020; Li et al. 2021; Gu et al. 2021). Therefore, in this study, we investigated whether METTL3 is involved in the progression of PE by affecting ER stress in trophoblasts.

YTH N^6^-methyladenosine RNA binding protein 2 (YTHDF2), the most efficient m^6^A reader, impairs the mRNA stability by recognizing m^6^A-containing mRNAs and assigning them to the processing bodies (Edupuganti et al. 2017). YTHDF2 plays an important role in several biological processes, such as the invasion, metastasis, proliferation, apoptosis, and differentiation of cells, in cancer and other diseases (Qiu et al. 2021; Hou et al. 2019, 2021; Yu et al. 2021). Although YTHDF2 greatly contributes to multiple biological and pathological processes, its role in PE under ER stress remains unclear.

In this study, we explored the m^6^A modification and the expression levels of METTL3 in PE placentas. Then, a model of ER stress was constructed with thapsigargin (TG) to mimic the ER stress observed in trophoblasts in vitro, and the relationship between m^6^A modification and METTL3 was analyzed. Furthermore, we explored the role of METTL3 in the regulation of ER stress by altering TMBIM6 expression in an m^6^A-YTHDF2-dependent manner in trophoblasts. Our study provided novel insights on PE pathogenesis.

## Methods

### Patient characteristics and placenta tissue collection

The study protocol was approved by the local ethics committee of Tongji Medical College, Huazhong University of Science and Technology. Informed consent was obtained from all 40 participants aged 22–41 years. PE was diagnosed according to the clinical criteria of the American College of Obstetricians and Gynecologists (ACOG Practice Bulletin No 2019). Exclusion criteria were as follows: (1) The healthy pregnant women with high blood pressure or proteinuria were excluded; (2) All pregnant women were excluded if they had a history of cardiovascular disease, diabetes, neurodegeneration, stroke, cancer, multiple pregnancies, placental abruption, or chorioamnionitis. Twenty healthy pregnant women were allocated to the healthy group, whereas the remaining preeclamptic women were allocated to the PE group. Their relevant clinical features are listed in the Table 1, and their placental specimens, which were approximately 2–3 cm from the edge of the umbilical cord attachment site (on the chorionic side), were collected. Each tissue sample was separated into two fractions: one for immunohistochemistry (IHC) and the other for RNA and protein extraction.

**Table 1:** Clinical characteristics of study population

Parameters	Healthy (n = 20)	Preeclampsia (n = 20)	p
Maternal age (year)	33.15 ± 0.985	31.4 ± 1.139	ns
Gestational age (week)	37.5 ± 0.716	35.15 ± 0.582	p < 0.05
Systolic blood pressure at delivery (mmHg)	112.75 ± 2.348	149.90 ± 2.021	p < 0.05
Diastolic blood pressure at delivery (mmHg)	72.40 ± 1.90	101.30 ± 1.24	p < 0.05
Proteinuria	0 (0/20)	20 (20/20)	p < 0.05
Body weight of infant (g)	3607.50 ± 57.50	2389.00 ± 134.93	p < 0.05

### Quantitative real-time PCR (qRT-PCR)

Total RNA was extracted from the collected placentas and cells in different groups using the TRNpure Total RNA Kit (HYCEZMBIO, China), and the concentration of RNA was quantified using Q5000 (QUAWELL, USA). A PrimeScript RT Reagent Kit (Takara, Tokyo, Japan) was used to synthesize the cDNA, and qRT-PCR was performed using a Step One Real-Time PCR System with ChamQ SYBR qPCR Master Mix (Vazyme, China), following the manufacturer’s instructions. The qRT-PCR reaction was conducted as follows: holding stage: 95 °C for 30 s, cycling stage: 40 cycles (95 °C for 10 s and 60 °C for 30 s), and melt curve stage: 95 °C for 15 s and 60 °C for 60 s. β-actin was used as an internal reference gene to quantify mRNA expression. Target mRNA expression levels were quantified according to the comparative cycle threshold (Ct; 2 − ΔΔCT) method, with β-actin as an internal reference gene. The qRT-PCR primer sequences used in this study are listed in Additional file 1: Table S1.

### m^6^A colorimetric assay

The m^6^A modification level in total RNA from placentas and cells was determined using the EpiQuik m^6^A RNA Methylation Quantification Kit (P-9005; Epigentek, USA), according to the manufacturer's protocol, after RNA quality analysis using Q5000 (QUAWELL, USA). Briefly, 200 ng RNA was added to the strip wells. The capture and detection antibody solutions were then added to the assay wells separately at appropriate dilution concentrations. m^6^A levels were colorimetrically quantified by measuring the absorbance at a wavelength of 450 nm. Finally, the data were calculated using relative quantification according to the manufacturer’s instructions.

### Immunohistochemistry

Placenta specimens were fixed with 4% paraformaldehyde. Servicebio Biotechnology Co., Ltd. (Wuhan, China) was employed for paraffin embedding, paraffin sectioning, and IHC staining. Primary antibodies against METTL3 (15073-1-AP, 1:300; Proteintech, China) and TMBIM6 (26782-1-AP, 1:300; Proteintech, China) were used. Each pair of placenta specimens from healthy and preeclamptic group was stained in the same condition. Sections of the immunohistochemical specimens were observed and imaged using a microscope (Olympus, Japan). Finally, Image Pro Plus6.0 was used to analyze the data. Specifically, the optical density (OD) of IHC images were firstly standardized and the positive areas were selected and measured to calculate the integrate OD (IOD) and mean IOD. Mean IOD was used for statistical analysis.

### Western blot (WB) analysis

Total protein was extracted from the cells and tissues using the RIPA lysis buffer (Servicebio, China), mixed with protease inhibitors (Beyotime, China), and the protein concentrations were quantified using a BCA protein assay kit (CWBIO, China). The protein samples were denatured at 95 °C for 10 min and stored at –80 °C until use. Equal amounts of proteins from different groups were separated by 10% SDS-PAGE gels and transferred onto PVDF membranes (0.45 mm pore size; Millipore, USA) which were then blocked with 5% skimmed milk in Tris-buffered saline containing 0.1% Tween-20 for 1 h. Next, the membranes were incubated with the METTL3 (15073-1-AP, 1:1000; Proteintech, China), TMBIM6 (26782-1-AP, 1:1000; Proteintech, China), GRP78 (11587-1-AP, 1:1000; Proteintech, China), CHOP (2895T, 1:1000; Cell Signaling Technology, USA), METTL14 (26,158–1-AP, 1:1000; Proteintech, China), fat mass and obesity-associated protein (FTO; 27226-1-AP, 1:1000; Proteintech, China), B-cell lymphoma-2 (Bcl-2; 26593-1-AP, 1:1500; Proteintech, China), Bcl-2-associated X (Bax; 2772T, 1:1000; Cell Signaling Technology, USA), heme oxygenase-1 (HO-1; 10701-1-AP, 1:3000; Proteintech, China), nuclear factor erythroid 2-related factor 2 (NRF2; 16396-1-AP; 1:1000; Proteintech, China), YTHDF2 (24744-1-AP, 1:4000; Proteintech, China), and β-actin (66009-1-Ig, 1:10,000; Proteintech, China) antibodies overnight at 4 °C. They were then washed thrice with TBST and incubated with the appropriate HRP-conjugated anti-rabbit or anti-mouse secondary antibodies (1:5000; Proteintech, China) at room temperature for 1 h. The proteins were visualized with enhanced chemiluminescence reagents (Millipore, USA), and the protein bands were analyzed using the ImageJ software.

### Cell culture and transfection

HTR-8/SVneo cell line, an extravillous trophoblastic cell line, was obtained from Dr. Charles Graham (Queen’s University, Canada) as a gift. Cells were cultured in RPMI 1640 medium (Biosharp, China) supplemented with 10% fetal bovine serum (Gibco, USA) and 100 U/mL penicillin & streptomycin (PYG0016, Boster, China) at 37 °C in an incubator containing 5% CO_2_. HTR-8/SVneo cells were cultured in a 6-well plate and transfected with Lipofectamine 3000 (Invitrogen, USA). A TMBIM6 overexpression model was established via TMBIM6 plasmid transfection for 24 h. Simultaneously, a negative control model was established via an empty vector transfection. Knockdown of METTL3 and YTHDF2 was achieved using three shRNAs targeting the *METTL3* and *YTHDF2* genes, which were synthesized by GenePharma Biotech. The target sequences are listed in Additional file 2: Table S2. Transfection efficiency was confirmed by qRT-PCR or WB. To construct a model of ER stress, HTR-8/SVneo cells were incubated with 100 nmol/L TG (T863962; Macklin Biochemical Co., Ltd, China) for 6 h after transfection with overexpression plasmids or shRNAs and incubated for the last 18 h. Finally, HTR-8/SVneo cells were collected to extract the RNAs and proteins for subsequent experiments.

### Methylated RNA immunoprecipitation (MeRIP)-qPCR

The m^6^A modification of TMBIM6 was determined using the Magna EpiQuik CUT&RUN m^6^A RNA Enrichment (MeRIP) Kit (P-9018, Epigentek, USA), according to the manufacturer’s instructions. Briefly, 50 μg of total RNA was used for m^6^A immunoprecipitation, and 1/10 of it was saved as the input control group. First, an immunocapture solution containing the m^6^A antibody, non-immune IgG, affinity beads, immunocapture buffer, and RNA sample was prepared and vortexed for 90 min at room temperature to immunocapture m^6^A RNA. Then, the cleavage enzyme mix was used to cleave RNA, and proteinase K and RNA purification solution were added to the immunoprecipitated complex to remove the excess proteins and purify m^6^A-containing RNA. Finally, the immunoprecipitated m^6^A RNA was recovered using the elution buffer, and its level was measured using qRT-PCR.

### Cell viability assay

Cell viability was assessed using the Cell Counting Kit-8 (CCK8; Proteintech, China). Approximately 0.6 × 10^4^ cells were seeded in each well of a 96-well plate and treated with TG at different concentrations (0, 25, 50, 100, 200 nmol/L) for 1, 6, 12, 24 h. Later, the CCK-8 reagent (10 μL) was added to each well, followed by 2 h incubation at 37 °C in a dark incubator. Absorbance was measured at 450 nm using a multimode reader (Infinite F50; Tecan).

### Measurement of ROS

HTR-8/SVneo cells were seeded in a 6-well plate and washed thrice with PBS after various treatments. Then, 2′-7′-dichlorodihydrofluorescein diacetate (DCFH-DA, CA1410; Solarbio, China) kit, one of the most widely used commodities to directly measure the intracellular ROS levels, was added to the plates and incubated for 20 min at 37 °C in a dark atmosphere. Finally, the green fluorescence indicating the level of intracellular ROS was observed by fluorescence microscopy (Olympus, Japan) at 100 ×, and the mean fluorescence intensity was calculated using the ImageJ software to determine the level of intracellular ROS.

### Flow cytometry analysis

To measure apoptosis, HTR-8/SVneo cells were harvested and stained using the Annexin V-FITC Apoptosis Detection Kit (KGA107, Keygen Biotech, China) for 10 min at room temperature in the dark. Flow cytometry was used to detect apoptosis, and the data were analyzed using the FlowJo 10.5.3 software.

### Transwell assays

Cell invasion was detected using Transwell chambers (8.0 μm pore size; Corning, USA) and Matrigel mix (BD Biosciences, CA, USA). Specifically, 3 × 10^4^ cells were resuspended in serum-free RPMI 1640 medium (200 μL) in the upper chamber, while the lower chamber contained 600 μL medium supplemented with 10% fetal bovine serum. The cells were incubated for 24 h at 5% CO_2_ and 37 °C, fixed with 4% paraformaldehyde for 20 min, and stained with crystal violet for 15 min. Finally, cells in the lower compartment of the chamber were counted under an inverted microscope (Olympus, Japan) at a magnification of 100 ×, and five random fields were selected for cell counting in each chamber.

### mRNA stability assay

After treatment with 5 μg/mL actinomycin D (7240-37-1; Macklin, China), which inhibits mRNA transcription, for 0, 2, 4, and 6 h, total RNA was extracted using a TRNpure Total RNA Kit (HYCEZMBIO, Wuhan, China). The mRNA level of TMBIM6 at the indicated time was measured via qRT-PCR, and the degradation percentage was calculated. The turnover rate and half-life of RNA were estimated according to a previously published method (Zhang et al. 2021a). The constant of RNA decay (K) was calculated using the following equation: dC/dt = − KC, where dC/dt is the change in RNA concentration at a given time and C is the RNA concentration. Subsequently, the equation ln(C/C0) = − Kt was used to estimate the K value which represents the degradation rate of RNA. RNA half-life time (t_1/2_), indicating that 50% of the RNA had decayed (C/C0 = 1/2), was also calculated based on the equation: t_1/2_ = ln2/K.

### Animals and experimental groups

The animal study was conducted with the approval of the Animal Ethics Committee of the Huazhong University of Science and Technology, and all experimental procedures were performed according to the National Institutes of Health Guidelines and Regulations. Twenty-four SD rats aged 8–10 weeks (240–260 g) were purchased from the experimental animal center of Sanxia University (Sanxia, China) and raised in the animal laboratory of Tongji Medical College. Each rat was housed under SPF conditions with water and food ad libitum. Female rats were copulated with weight-matched male rats in a ratio of 2:1. The first gestational day (GD1) of pregnancy was defined as when the sperm or vaginal suppositories were observed under a light microscope, as shown in Fig. 8B. The time scheme of the animal experiment is shown in a schematic diagram (Fig. 8A). Pregnant female rats (n = 24) were randomly divided into healthy (n = 6) and disease (n = 18) groups, and the disease groups (n = 18) were further divided into three subgroups: PE, PE + LV-sh-Con, and PE + LV-sh-METTL3 groups. L-nitro-arginine methyl ester (L-NAME; R015327; RHAWN, China), a NOS inhibitor, was used to establish the PE model, as previously reported (Burke et al. 2016). The healthy group (NC, n = 6) received an intraperitoneal injection of sterile 0.9% NaCl, whereas the three disease subgroups were injected with L-NAME (50 mg/kg/day) between GD10 and GD15. Next, the PE + LV-sh-Con group was injected with a lentivirus containing sh-Con RNA (LV-sh-Con), whereas the PE + LV-sh-METTL3 group was injected with an equal dose of lentivirus containing METTL3 shRNA (LV-sh-METTL3) on GD15. The transfection efficiency of LV-shRNA in rats was ascertained by observing the expression of GFP in frozen placental sections under a fluorescence microscope (Fig. 8M). Then, the rats were anesthetized using 10% chloral hydrate (Macklin, China), and the embryonic, placental, and kidney samples were immediately removed by cesarean section on GD20. Finally, the fetuses and placentas were weighed, and placental samples were collected for WB and staining.

### Haematoxylin‐eosin (HE) staining

The paraffin‐embedded kidney tissues were sliced to serial sections of 5-μm thickness. Subsequently, the slices were depafaffinized, debenzolized, and conventionally stained with HE. After staining was completed, the sample slides were mounted using neutral balsam and sealed with clean coverslips. Finally, the sections were photographed, and histopathological changes were observed under a microscope.

### Blood pressure and urinary protein analysis

Systolic blood pressure (SBP) was monitored using a non-invasive blood pressure system (Kent Scientific, USA) on GD9, GD15, and GD20. Each rat was preheated to 38 °C for 5 min before each measurement. Urinary protein levels were determined in the urine samples using CBB kits (Jiancheng Institute of Biotechnology, China) on GD9, GD15, and GD20. Blood pressure and urinary protein levels on GD9 are shown in Additional file 3: Table S3. Increase in blood pressure to > 30 mmHg and significant increase in urinary protein levels indicated the successfully establishment of the PE rat model.

### Statistical analysis

Statistical analysis was performed using the GraphPad Prism8.0 and SPSS 20.0 software. The results are presented as the mean ± SEM. Two-group comparisons were analyzed using Student’s t-test, while three or more group comparisons were carried out using one-way ANOVA. p-values less than 0.05 (p < 0.05) were considered to be statistically significant.

## Results

### METTL3 expression and m^6^A levels were increased in preeclamptic placentas

**Fig. 1:**
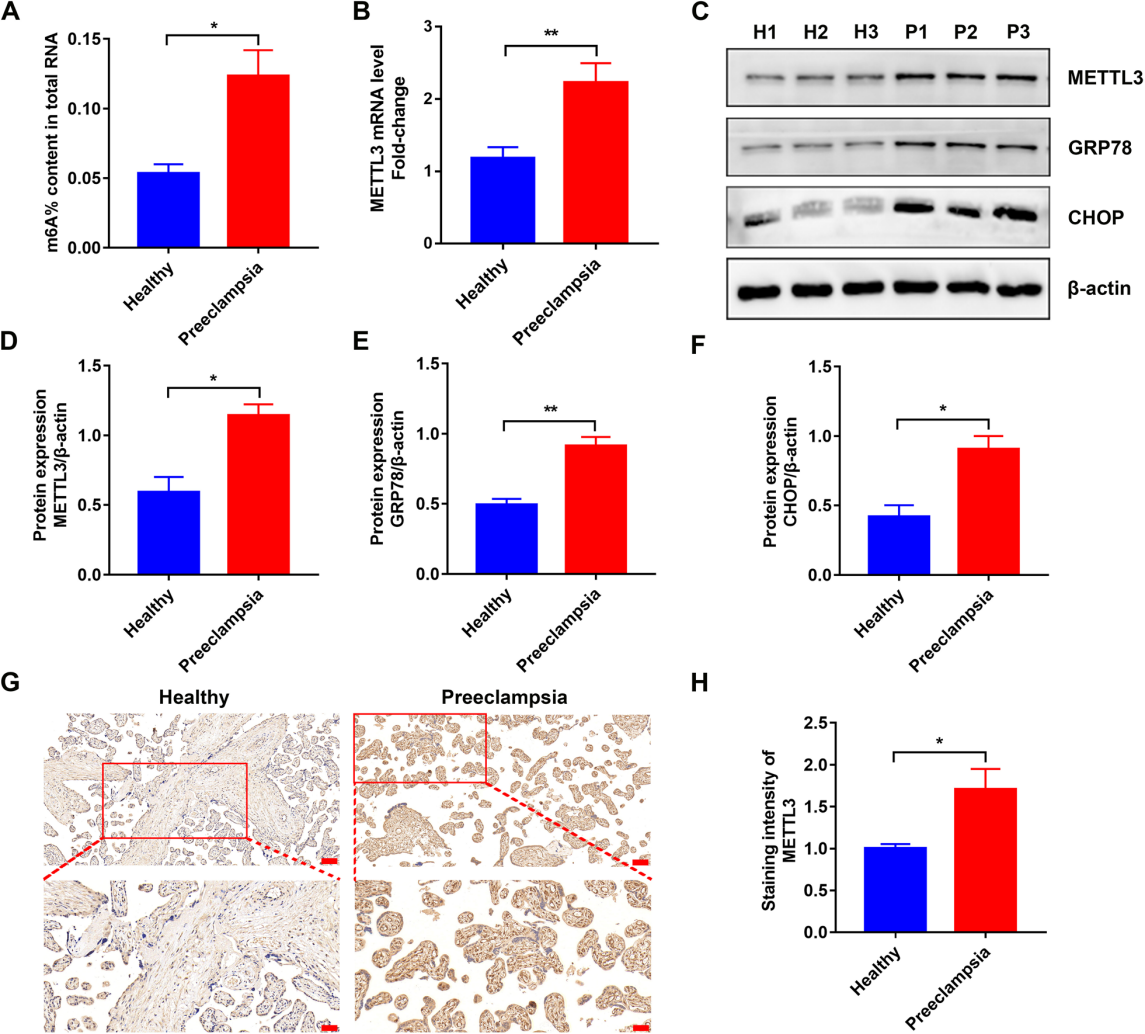
Different METTL3 expression and m^6^A levels in the placentas from preeclamptic and healthy pregnant women. **A** The different m^6^A levels determined by m^6^A colorimetric assay. **B** The different METTL3 mRNA levels detected by qRT-PCR. **C**–**F** Representative WB images and densitometry quantification of METTL3, GRP78 and CHOP. The data were normalized to β-actin. **G** METTL3 expression determined by IHC. Photographs were obtained at 100 × (up, *Scale bar* 100 μm) and 200 × (down, *Scale bar* 50 μm) magnification. **H** Qualified data shown in **G**. H Healthy Pregnant Women; P: Preeclamptic Pregnant Women. n = 3. ns: no significance, *p < 0.05, **p < 0.01, ***p < 0.001

Existing RNA-Seq and MeRIP-Seq data have demonstrated that m^6^A modification of mRNAs may contribute to PE (Wang et al. 2020; Taniguchi et al. 2020). To investigate the role of the m^6^A modification in PE, we measured the relative levels of m^6^A in 20 pairs of placental tissues from preeclamptic and healthy pregnant women using the colorimetric method. The colorimetric results indicated that m^6^A levels were significantly upregulated in preeclamptic placentas compared to healthy placentas (Fig. 1A). METTL3 is the most well-known m^6^A methyltransferase that mediates reversible m^6^A modification. Therefore, we measured its expression levels in placental tissues via qRT-PCR. The results showed that the mRNA levels of METTL3 were significantly upregulated in PE (Fig. 1B), which was consistent with m^6^A alteration. WB (Fig. 1C, D) and IHC (Fig. 1G, H) results revealed that the protein expression of METTL3 showed a similar trend. These results suggest that METTL3 might contribute to PE. Interestingly, the levels of two ER stress-associated markers, CHOP and GRP78, were significantly increased in preeclamptic placentas (Fig. 1C, E, F), which was consistent with previous studies (Verma et al. 2018; Du et al. 2017). Thus, it was reasonable to assume that the detrimental ER stress response was involved in PE.

### METTL3 expression and m^6^A levels were upregulated in TG-induced ER stress model

**Fig. 2:**
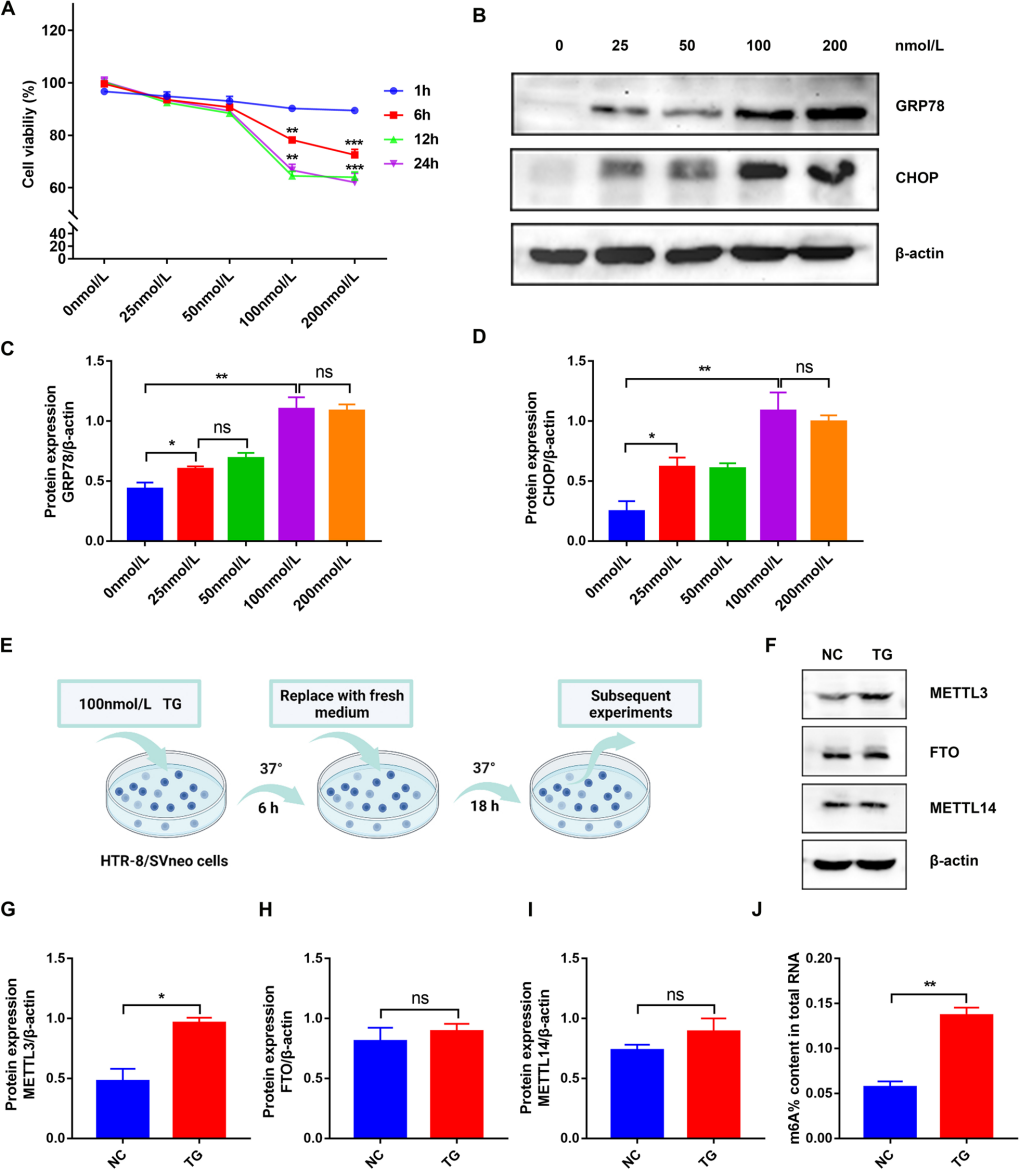
Construction of ER stress model in HTR-8/SVneo cells where METTL3 expression and m^6^A level were upregulated. **A** Cell viability determined by the CCK8 analysis. **B**–**D** Representative WB images and densitometry quantification of GRP78 and CHOP. The data were normalized to β-actin. **E** Flow diagram of constructing ER stress model. HTR-8/SVneo cells were treated for 6 h with 100 nmol/L TG and another 18 h with fresh medium and used for the following experiments. **F**–**I** Representative WB images and densitometry quantification of METTL3, FTO and METTL14. The data were normalized to β-actin. **J** The m^6^A levels determined by m^6^A colorimetric assay. n = 3. ns: no significance, *p < 0.05, **p < 0.01, ***p < 0.001

To further investigate the relationship between ER stress and PE, a cell model was constructed using TG, an inhibitor of sarco-endoplasmic reticulum Ca^2+^-ATPases, to induce ER stress (Jaskulska et al. 2020). First, HTR-8/SVneo cells were exposed to 0, 25, 50, 100, and 200 nmol/L TG for 1, 6, 12, and 24 h, and the CCK-8 assay showed that TG significantly reduced cell viability at concentrations of 100 or 200 nmol/L, and the culture time lasted for 6, 12, or 24 h (Fig. 2A). Interestingly, many HTR-8/SVneo cells died when treated with 100 or 200 nmol/L TG for 12 or 24 h. Later, WB revealed that the expression of GRP78 and CHOP peaked when the concentration reached 100 nmol/L, and there was no significant difference between 100 and 200 nmol/L (Fig. 2B–D). Therefore, 100 nmol/L TG treatment with 6 h of culture was chosen for the following experiments (Fig. 2E). Considering that METTL14, another important methyltransferase for m^6^A modification (Zhou et al. 2021), and FTO, a main type of demethylase (Bartosovic et al. 2017) were another two main enzymes affecting m^6^A modification, WB was conducted to explore the expression of these three significant enzymes. Interestingly, the results indicated that METTL3 was significantly upregulated, while METTL14 and FTO showed no difference in TG-induced HTR-8/SVneo cells (Fig. 2F–I). Meanwhile, the colorimetric results showed increased m^6^A modification in treated cells, which was consistent with the results in placental tissues (Fig. 2J). Thus, METTL3 was selected for subsequent experiments to explore the m^6^A modification in PE.

### Knockdown of METTL3 attenuated ER stress, decreased ROS production and apoptosis rate in TG-treated HTR-8/SVneo cells

**Fig. 3:**
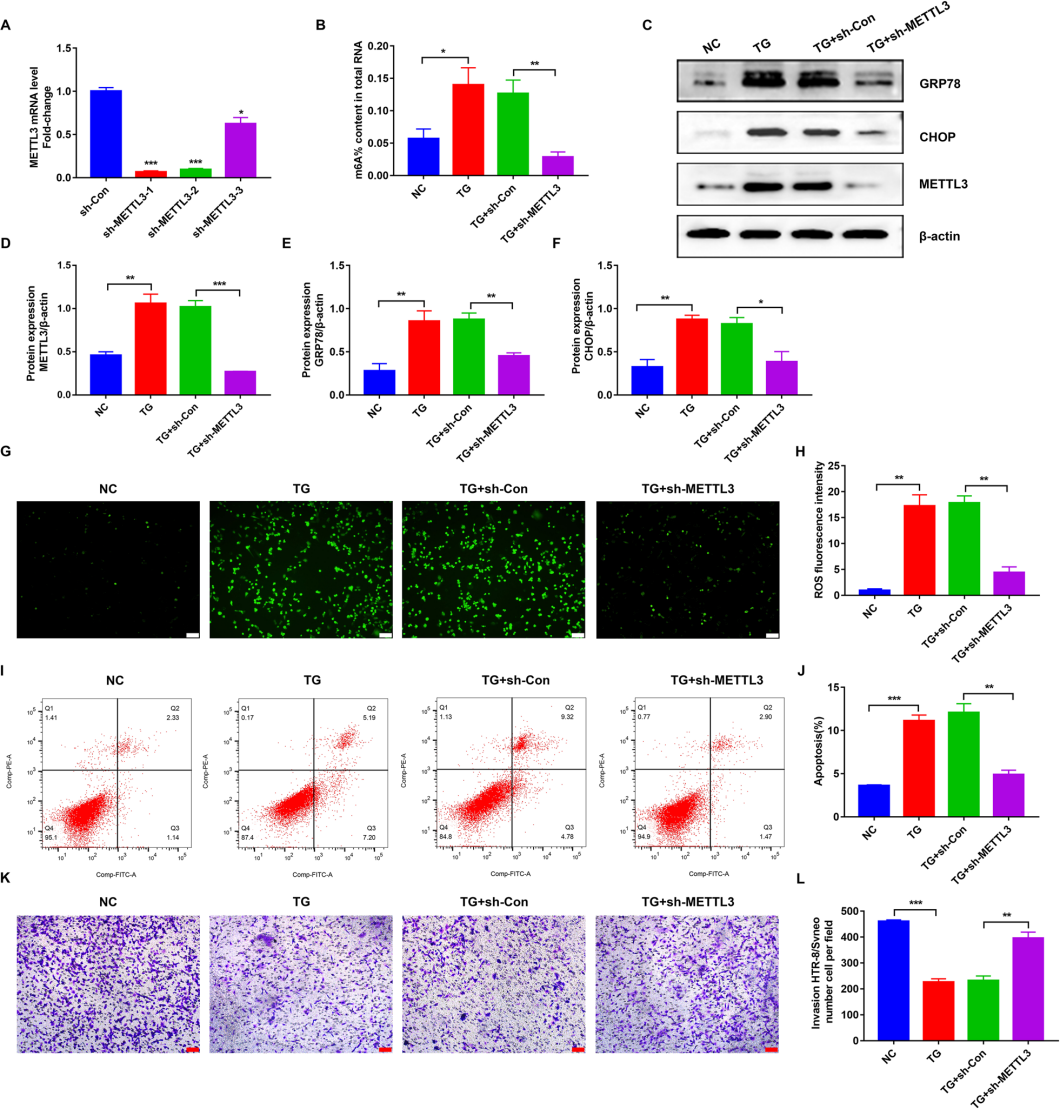
Attenuated ER stress, decreased ROS production and apoptosis rate in TG-treated HTR-8/SVneo cells by knockdown of METTL3. **A** The knockdown efficiency of different sh-METTL3s determined by qRT-PCR. **B** The m^6^A level in different groups determined by m^6^A colorimetric assay. **C**–**F** Representative WB images and densitometry quantification of METTL3, GRP78 and CHOP. The data were normalized to β-actin. **G**, **H** ROS production detected by DCFH-DA. Photographs were obtained at 100 × (*Scale bar* 100 μm). **I**, **J** Apoptosis rate in different groups detected by flow cytometry. **K** Representative transwell photos of HTR-8/SVneo cells in different groups (100 × magnification, *Scale bar* 100 μm). **L** Qualified data shown in **K**. n = 3. *p < 0.05, **p < 0.01, ***p < 0.001

To further explore the role of METTL3 in PE, we established METTL3 knockdown models with three independent shRNA sequences (sh-METTL3-1, 2 and 3) in HTR-8/SVneo cells, and qRT-PCR confirmed successful knockdown (Fig. 3A). Furthermore, the colorimetric results showed downregulation of m^6^A modification, as METTL3 decreased in TG-induced HTR-8/SVneo cells (Fig. 3B). Meanwhile, the ER stress state improved, as indicated by the decreased expression of GRP78 and CHOP (Fig. 3C–F). Immunofluorescence and flow cytometry showed that knockdown of METTL3 could reduce ROS production and apoptotic rate which were elevated in the ER stress state (Fig. 3G–J), and this can be explained by the theory that ER stress was able to induce apoptosis through an ROS-dependent pathway (Zeeshan et al. 2016). Moreover, Transwell assays indicated that the impaired invasion ability of HTR-8/SVneo cells caused by TG was restored by the knockdown of METTL3 (Fig. 3K–L). Taken together, these results indicate that METTL3 knockdown could maintain ER homeostasis during ER stress, reduce ROS production and apoptosis, and increase the invasion of TG-induced HTR-8/SVneo cells.

### METTL3 regulated the ER stress via TMBIM6 in HTR-8/SVneo cells

**Fig. 4:**
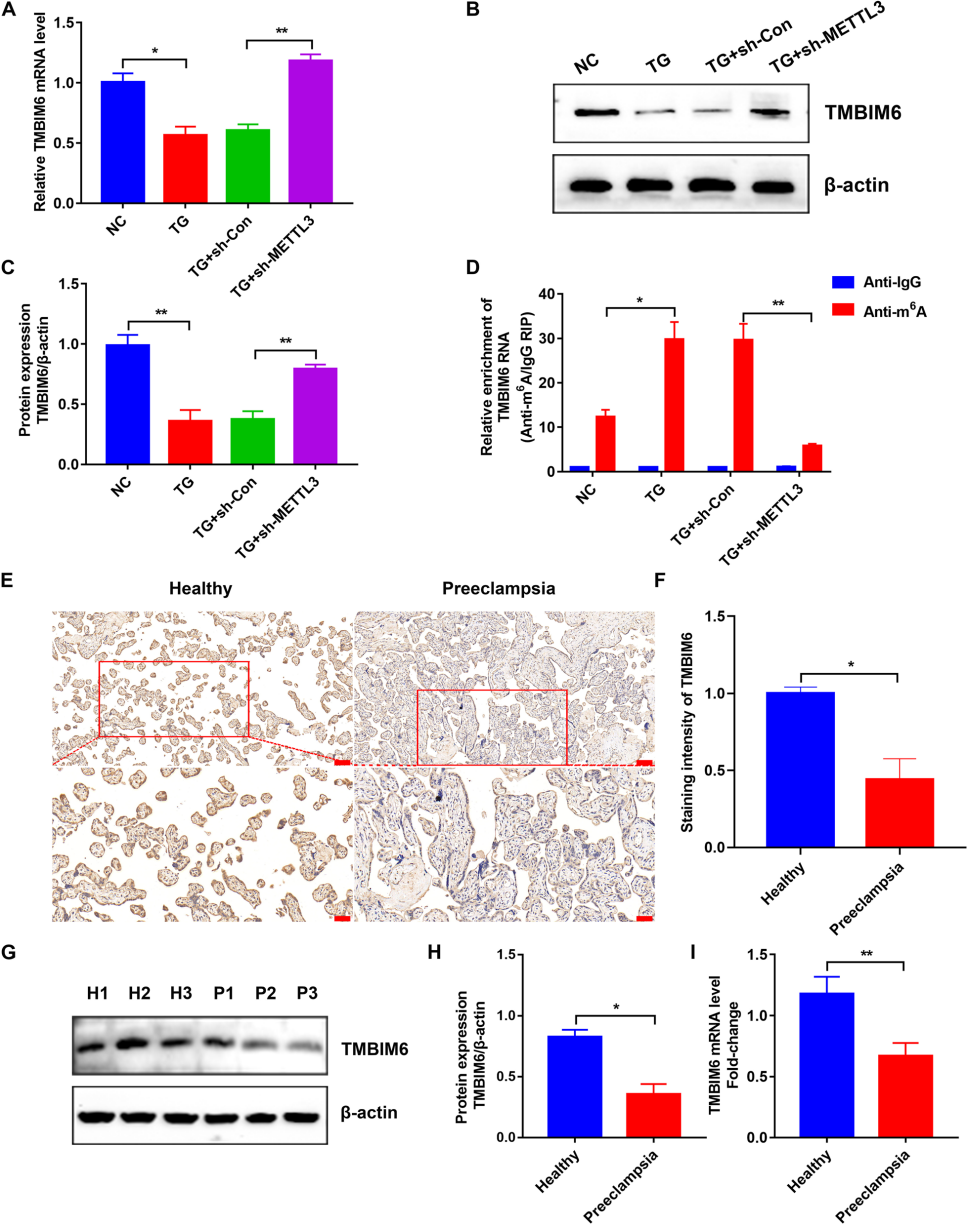
The relationship between METTL3 and TMBIM6 in HTR-8/SVneo cells under ER stress. **A** The relative expression levels of TMBIM6 mRNA in different groups detected by qRT-PCR. **B**, **C** Representative WB images and densitometry quantification of TMBIM6 in different groups. The data were normalized to β-actin. **D** The m^6^A level of TMBIM6 mRNA in different groups measured by MeRIP-qPCR. **E** TMBIM6 expression determined by IHC. Photographs were obtained at 100 × (up, *Scale bar* 100 μm) and 200 × (down, *Scale bar* 50 μm) magnification. **F** Qualified data shown in **E**. **G, H** Representative WB images and densitometry quantification of TMBIM6 in the placentas from preeclamptic and healthy pregnant women. The data were normalized to β-actin. **I** The relative expression levels of the TMBIM6 mRNA in healthy and preeclamptic placentas determined by qRT-PCR. H: Healthy Pregnant Women; P: Preeclamptic Pregnant Women. n = 3. *p < 0.05, **p < 0.01

Previous studies have shown that TMBIM6 is essential for ER homeostasis (Lebeaupin et al. 2020; Bhattarai et al. 2021). The m^6^A sites of TMBIM6 were predicted using two databases, RMBase v2.0 (http://rna.sysu.edu.cn/rmbase/) and M6A2Target (http://m6a2target.canceromics.org/) (Deng et al. 2021; Xuan et al. 2018). The prediction showed that TMBIM6 mRNA contained m^6^A sites and could be modified by METLL3 (Additional file 4: Fig. S1, Additional file 5: Fig. S2). Based on the above results, we hypothesized that this protein was associated with METTL3 in TG-induced HTR-8/SVneo cells. Next, qRT-PCR and WB were performed to explore the expression of TMBIM6. Interestingly, in TG-induced HTR-8/SVneo cells, TMBIM6 was significantly decreased at both the mRNA and protein levels, whereas knockdown of METTL3 could reverse this change (Fig. 4A–C). Moreover, MeRIP-PCR showed a similar trend in m^6^A levels of TMBIM6 (Fig. 4D). We further hypothesized that METTL3 could affect ER stress and cellular functions via TMBIM6 in HTR-8/SVneo cells, which was related to PE. IHC indicated that TMBIM6 was significantly downregulated in preeclamptic placentas (Fig. 4E, F), which was further validated by WB (Fig. 4G, H) and qRT-PCR (Fig. 4I). As expected, TMBIM6 mRNA and protein levels were significantly decreased in preeclamptic placentas. In conclusion, these results indicated that METTL3 could affect ER stress and cellular functions via TMBIM6, resulting in PE.

### Overexpression of TMBIM6 attenuated ER stress, decreased ROS production and apoptosis rate in TG-treated HTR-8/SVneo cells

**Fig. 5:**
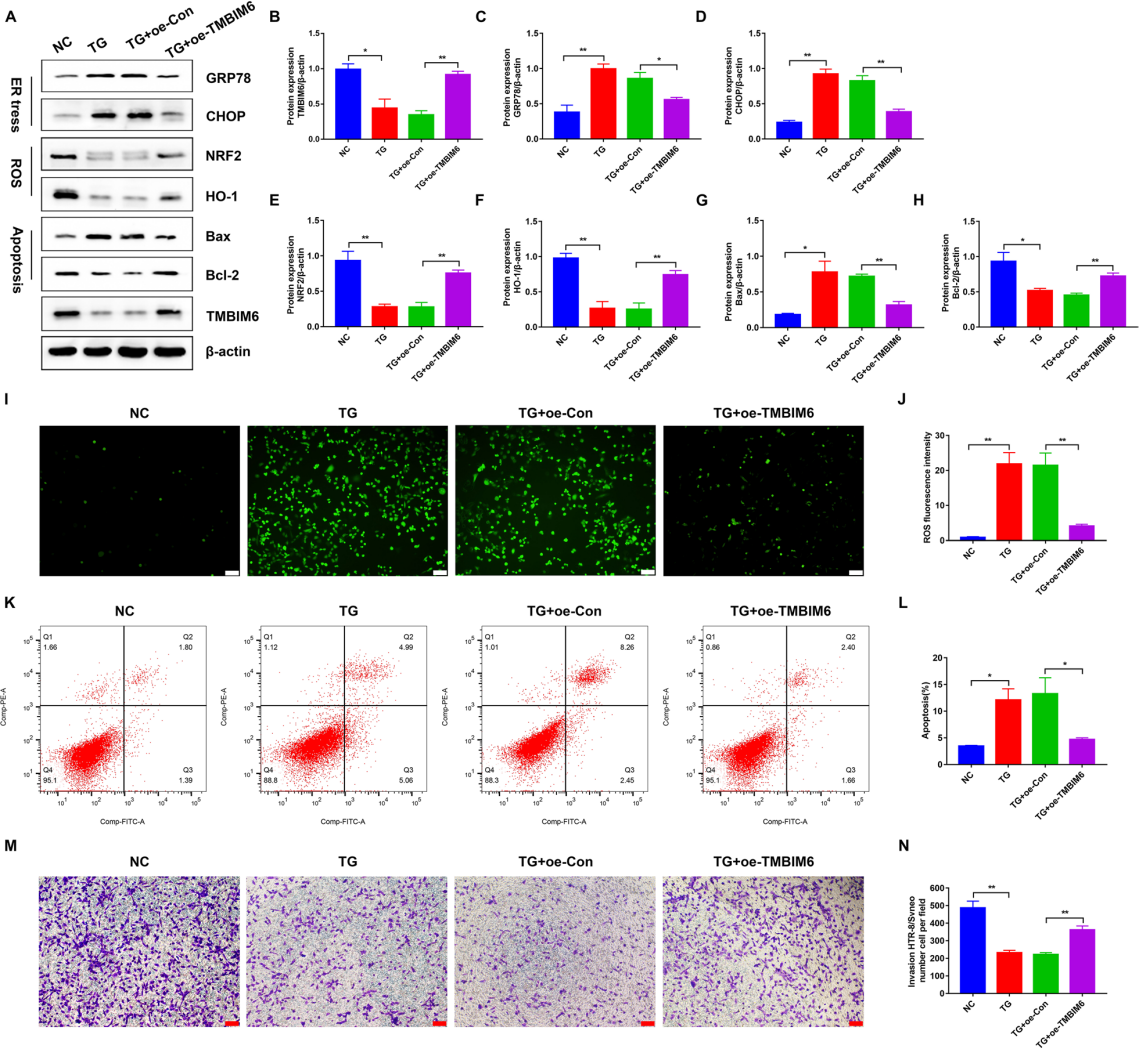
Attenuated TG-induced ER stress, oxidative stress and apoptosis in HTR-8/SVneo cells by overexpressing TMBIM6. **A**–**H** Representative WB images and densitometry quantification of TMBIM6, GRP78, CHOP, NRF2, HO-1, Bax and Bcl-2 in different groups. The data were normalized to β-actin. **I**, **J** ROS production detected by DCFH-DA. Photographs were obtained at 100 × (*Scale bar* 100 μm). **K**, **L** Apoptosis rate in different groups detected by flow cytometry. **M** Representative transwell photos of HTR-8/SVneo cells in different groups (100 × magnification, *Scale bar* 100 μm). **N** Qualified data shown in M. Oe-Con: Overexpression of control plasmid; Oe-TMBIM6: Overexpression of TMBIM6 plasmid. n = 3. *p < 0.05, **p < 0.01

To explore the association between METTL3 and TMBIM6, we first determined the role of TMBIM6 in PE. The model of ER stress was used, and overexpression of TMBIM6 was obtained in TG-induced HTR-8/SVneo cells. As expected, ER stress was significantly suppressed with TMBIM6 increased, as indicated by the downregulated expression of GRP78 and CHOP (Fig. 5A–D). Meanwhile, the flow cytometry and immunofluorescence showed that the apoptotic rate and ROS production were significantly decreased with two apoptosis-related markers, Bcl-2 upregulated and Bax downregulated and two antioxidant factors, NRF2 and HO-1, upregulated when TMBIM6 was overexpressed in TG-induced HTR-8/SVneo cells (Fig. 5A, E–L). Moreover, transwell assays indicated that overexpression of TMBIM6 also increased the invasion ability of TG-induced HTR-8/SVneo cells (Fig. 5M, N). In brief, these results indicated that TMBIM6 could maintain ER homeostasis during ER stress, reduce ROS production and apoptosis, and also increase the invasive ability of TG-induced HTR-8/SVneo cells.

### TMBIM6 acted as a downstream target of METTL3 under ER stress, oxidative stress, and apoptosis in HTR-8/SVneo cells

**Fig. 6:**
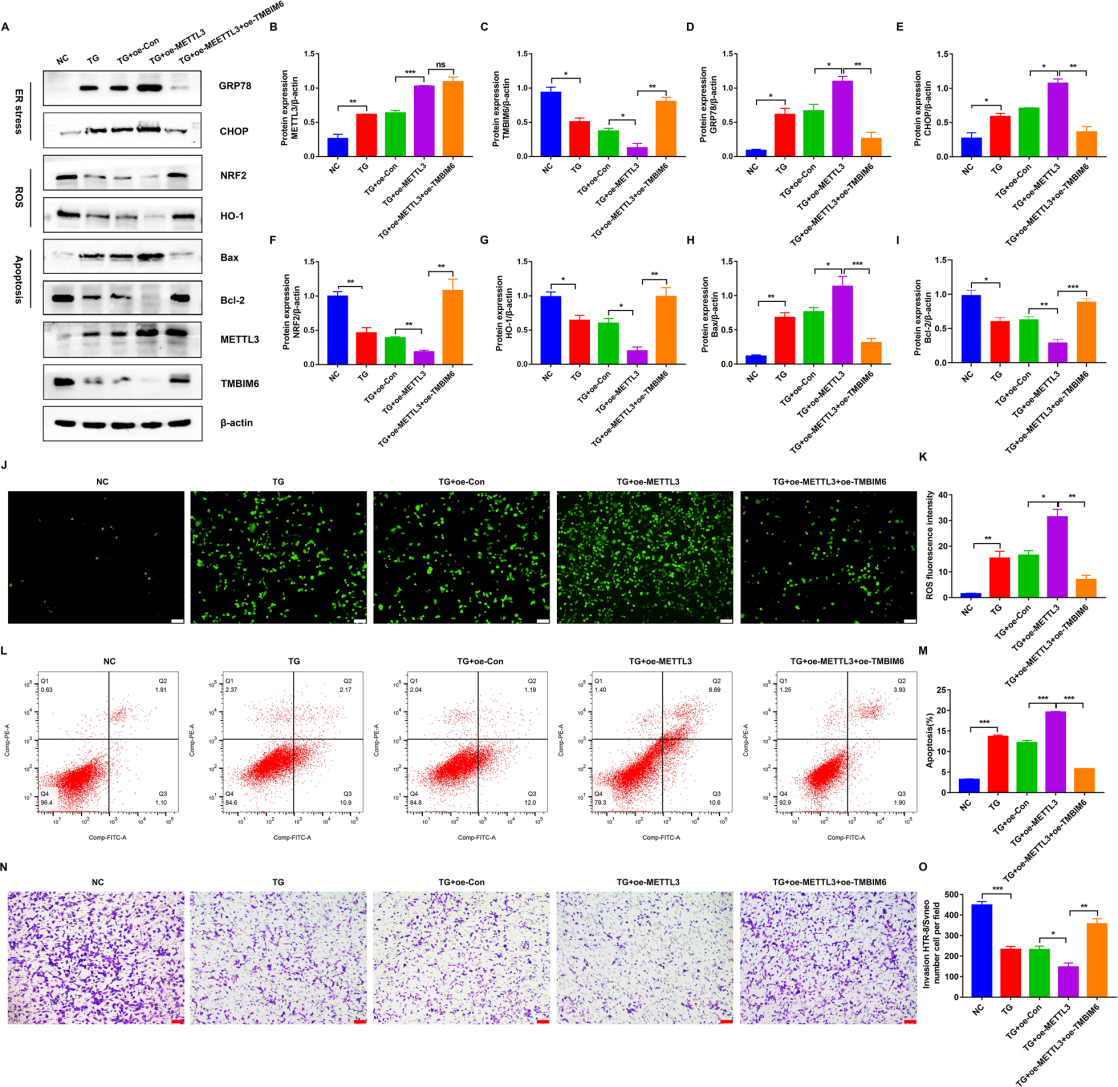
Overexpression of TMBIM6 partially neutralized the effects of METTL3 on TG-induced ER stress, oxidative stress and apoptosis in HTR-8/SVneo cells. **A**–**I** Representative WB images and densitometry quantification of METTL3, TMBIM6, GRP78, CHOP, NRF2, HO-1, Bax and Bcl-2 in different groups. The data were normalized to β-actin. **J**, **K** ROS production detected by DCFH-DA. Photographs were obtained at 100 × (*Scale bar* 100 μm). **L**, **M** Apoptosis rate in different groups detected by flow cytometry. **N** Representative transwell photos of HTR-8/SVneo cells in different groups (100 × magnification, *Scale bar* 100 μm). **O** Qualified data shown in **L**. Oe-Con: Overexpression of control plasmid; Oe-METTL3: Overexpression of METTL3 plasmid; Oe-TMBIM6: Overexpression of TMBIM6 plasmid. n = 3. *p < 0.05, **p < 0.01, ***p < 0.001

Based on the functions of TMBIM6 and its association with METTL3, we reasonably supposed that TMBIM6 could be a downstream regulator of METTL3. To test this further, we simultaneously overexpressed METTL3 and TMBIM6. Contrary to the knockdown of METTL3, its overexpression significantly aggravated the ER stress state in TG-induced HTR-8/SVneo cells, as indicated by the upregulation of GRP78 and CHOP in WB (Fig. 6A–E). However, this state was improved by the overexpression of TMBIM6. Meanwhile, the intracellular ROS and the expression of NRF2, HO-1, Bax, and Bcl-2 were measured (Fig. 6A, F–K), and flow cytometry and transwell assays were conducted (Fig. 6L–O). Interestingly, similar trends were observed in ROS production, apoptotic rate, and invasion ability of TG-induced HTR-8/SVneo cells. These results indicated that TMBIM6 could partially neutralize the negative effects of METTL3 and mediate its functions in TG-induced HTR-8/SVneo cells. Therefore, it was reasonable to consider TMBIM6 as a downstream regulator of METTL3.

### METTL3 affected the expression of TMBIM6 by regulating the TMBIM6 mRNA stability via YTHDF2

**Fig. 7:**
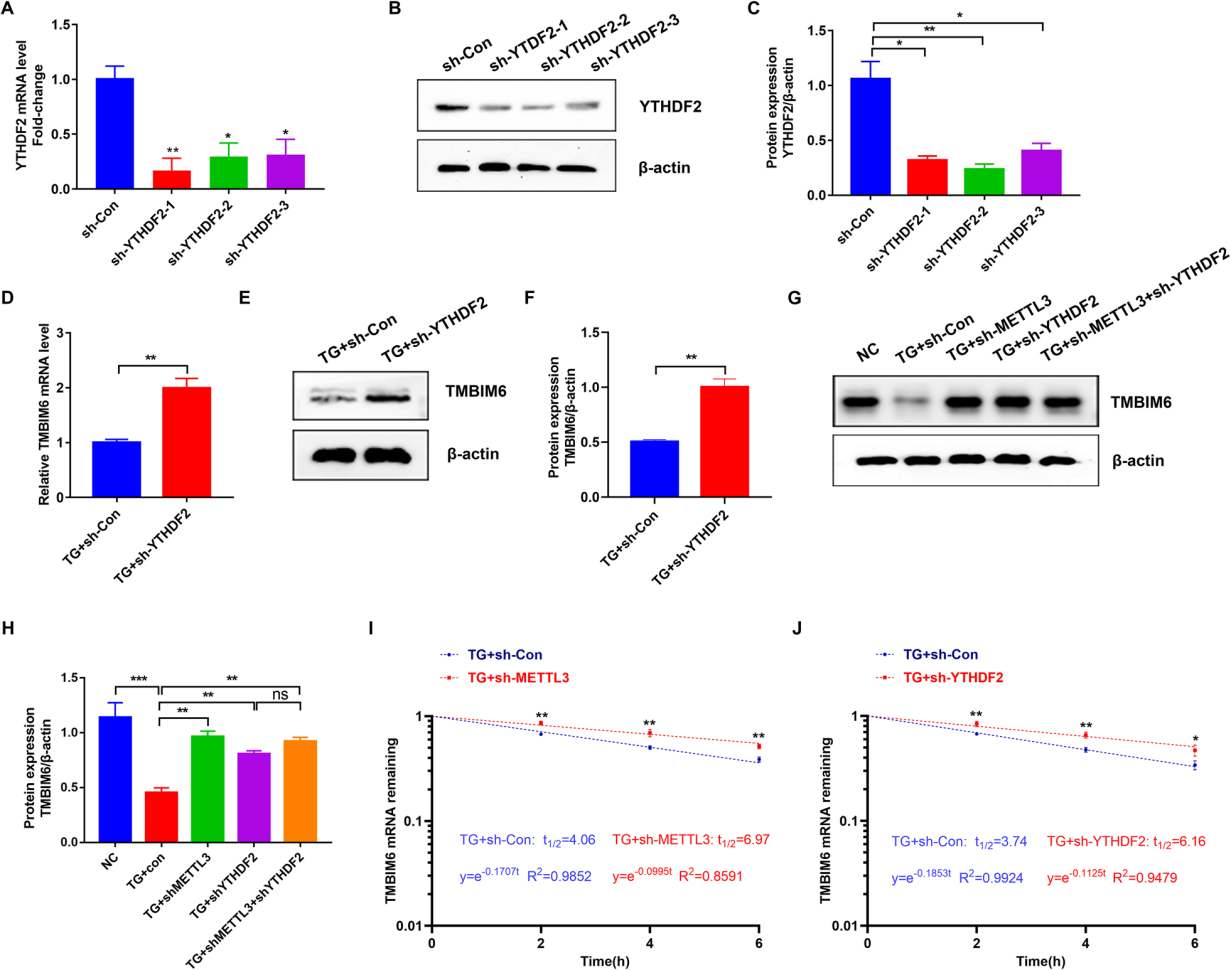
METTL3 affected the expression of TMBIM6 by regulating TMBIM6 mRNA stability via YTHDF2 involvement. **A**–**C** The knockdown efficiency of different sh-YTHDF2s determined by qRT-PCR and WB. **D** The relative expression levels of the TMBIM6 mRNA in sh-Con and sh-YTHDF2 group determined by qRT-PCR. **E**–**H** Representative WB images and densitometry quantification of TMBIM6 in different groups. The data were normalized to β-actin. **I**, **J** Transcript stability assay of TMBIM6 in different groups. n = 3. ns: no significance, *p < 0.05, **p < 0.01, ***p < 0.001

Present results indicated that TMBIM6 was the target of METTL3. However, the further mechanism about how METTL3 inhibited TMBIM6 expression was still unclear. Since the m^6^A “reader” proteins could recognize methylated RNA and regulate its stability, we hypothesized that METTL3 could modify TMBIM6 mRNA by transferring methyl groups to m^6^A sites, and the m^6^A modification was identified by YTHDF2 mediating the degradation of TMBIM6 mRNA. We established YTHDF2 knockdown models with three independent shRNA sequences (sh-YTHDF2-1, 2, and 3) in HTR-8/SVneo cells, and qRT-PCR and WB confirmed successful knockdown (Fig. 7A–C, p < 0.05). First, WB and qRT-PCR showed that the expression of TMBIM6 was upregulated in TG-induced HTR-8/Svneo cells when YTHDF2 was downregulated (Fig. 7D–F). This implied that there was a relationship between TMBIM6 and YTHDF2. Later, we decreased the expression of METTL3 and YTHDF2 using sh-METTL3 and sh-YTHDF2 RNA in TG-induced HTR-8/SVneo cells, respectively, and a similar increase was observed in the expression of TMBIM6 (Fig. 7G, H). Meanwhile, RNA stability assays showed both knockdown of METTL3 and knockdown of YTHDF2 prolonged the half-life of TMBIM6 mRNA (Fig. 7I, J). Therefore, we hypothesized that METTL3 affected TMBIM6 through YTHDF2. Simultaneously, WB also indicated that there was no difference in the expression of TMBIM6 between knockdown of METTL3 and knockdown of both METTL3 and YTHDF2 (Fig. 7G, H). This implied that YTHDF2 had no effect when METTL3 was downregulated in TG-induced HTR-8/SVneo cells. In conclusion, these results indicated that YTHDF2 could recognize methylated TMBIM6 mRNA by METTL3 and decrease its stability, contributing to the dysfunction of HTR-8/SVneo cells.

### Knockdown of METTL3 improved systemic symptoms and protected the placentas and fetuses from PE in vivo

**Fig. 8:**
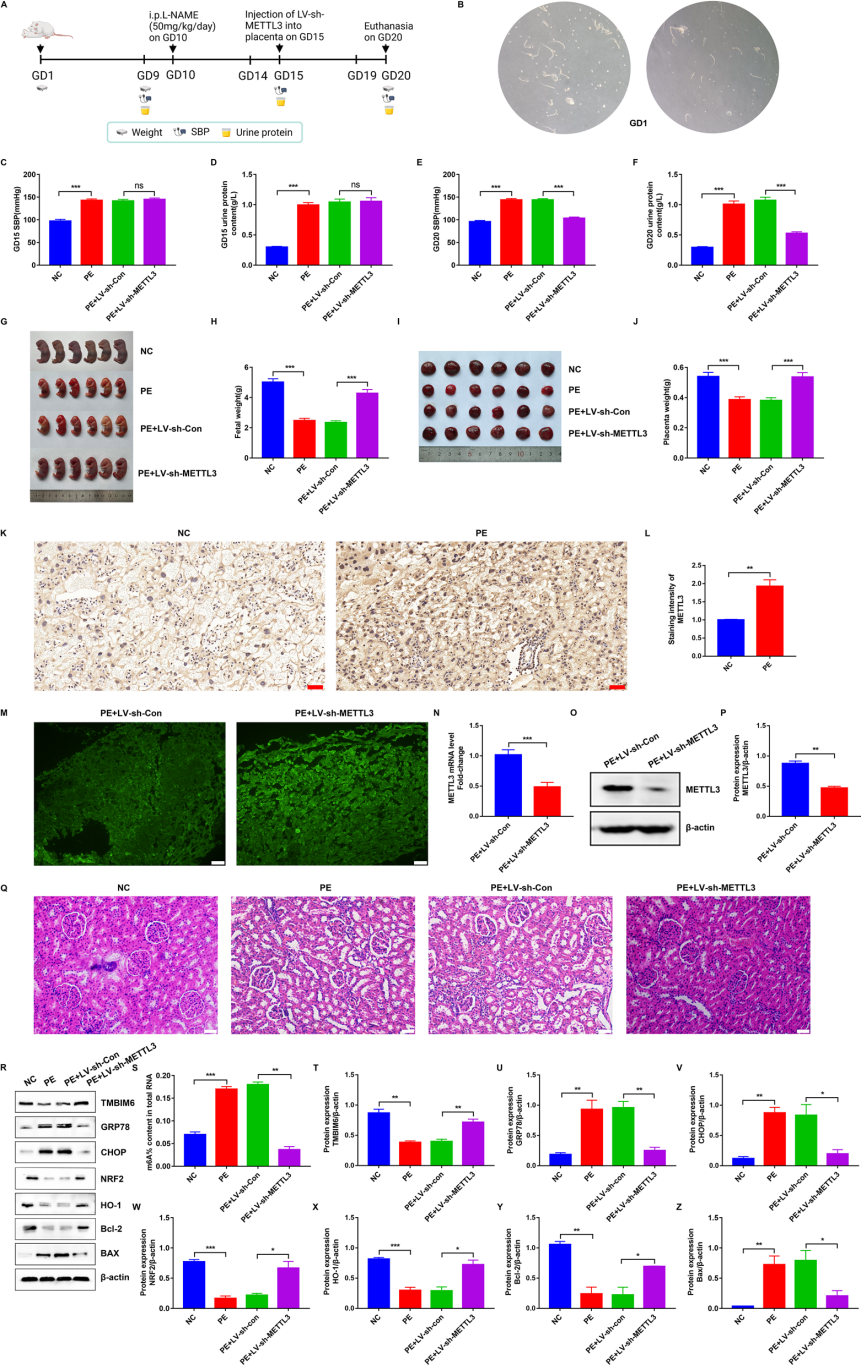
The therapeutic effect of inhibiting METTL3 in preeclamptic rat. **A** The flow diagram of our animal experiments. **B** The sperm harvested from the vagina of pregnant rats and observed under a light microscope on GD1. **C**, **E** The SBP measured on GD15 and GD20. **D**, **F** The urinary protein measured on the GD15 and GD20. **G**, **H** The photos of fetuses and the fetal weight on GD20 in different groups. **I**, **J** The photos of placentas and the placental weight on GD20 in different groups. **K** METTL3 expression in the placentas from preeclamptic and healthy pregnant rats determined by IHC. Photographs were obtained at 200 × (*Scale bar* 50 μm) magnification. **L** Qualified data shown in **K**. **M** The successful transfection into placentas with LV containing METTL3 shRNA indicated by the green fluorescence. Photographs were obtained at 100 × (*Scale bar* 100 μm) magnification. **N**–**P** The knockdown efficiency of METTL3 shRNA measured by qRT-PCR and WB. **Q** The pathological changes of kidneys from different groups indicated by HE staining. Photographs were obtained at 200 × (Scale bar 50 μm) magnification. **S** The different m^6^A levels in the placentas from different groups determined by m^6^A colorimetric assay. **R**, **T**–**Z** Representative WB images and densitometry quantification of TMBIM6, GRP78, CHOP, NRF2, HO-1, Bcl-2 and Bax in different groups. The data were normalized to β-actin. LV-sh-Con: lentivirus containing control shRNA; LV-sh-METTL3: lentivirus containing METTL3 shRNA. n = 3. ns: no significance, *p < 0.05, **p < 0.01, ***p < 0.001

To explore the therapeutic effect of METTL3, a preeclamptic rat model was first constructed, and IHC showed that METTL3 expression was upregulated in PE rats (Fig. 8K–L), which was consistent with preeclamptic pregnant women. Subsequently, we used a lentiviral vector encoding METTL3-targeting shRNA (LV-sh-METTL3) to knock METTL3 down in PE rats on GD15. The green fluorescence and downregulated METTL3 expression in the injected placentas demonstrated successful transfection by LV encoding sh-METTL3 (Fig. 8M–P). Interestingly, compared with controls, PE rats exhibited significant high SBP and obvious proteinuria that improved after administration of METTL3 shRNA on GD20 (Fig. 8C–F). After euthanasia, fetal rats, placentas, and kidneys from the parents were harvested and compared. METTL3 shRNA reversed PE-induced reductions in fetal weight and placental weight (Fig. 8G–J). Moreover, the kidneys from PE rats exhibited glomerular atrophying and renal tubular lumen enlargement, as indicated by HE staining, while METTL3 shRNA reversed these pathological changes (Fig. 8Q). Next, the colorimetric results showed upregulated m^6^A modification in the placentas from PE rats, which was reversed by METTL3 knockdown (Fig. 8S). Meanwhile, WB showed that the PE rat placentas were under severe ER stress, as indicated by the significantly upregulated GRP78 and CHOP, and the ROS production and apoptotic cells in them were also increased with NRF2, HO-1, and Bcl-2 downregulation and Bax upregulation (Fig. 8R, U–Z). Notably, METTL13 shRNA treatment improved the state of ER stress and antagonized PE-induced upregulation of ROS production and apoptotic cells by increasing TMBIM6 expression in preeclamptic placentas (Fig. 8R, T). Overall, these results indicated that the inhibition of METTL3 improved PE in PE rats.

## Discussion

Up to now, PE has been an intractable complication associated with pregnancy and brought about increasing mortality in both pregnant women and fetuses (Mol et al. 2016). Previous studies indicated that PE was always accompanied by trophoblast dysfunction due to ER stress, increased ROS production, and apoptosis rate (Capatina et al. 2021), and m^6^A modification could play an important role in this dysfunction (Taniguchi et al. 2020; Zhang et al. 2021b). While aberrant m^6^A modification for trophoblast dysfunction during PE has been observed (Wang et al. 2020; Gu et al. 2021; Guo et al. 2022), there are no reports on the relationship between the ER stress state in trophoblasts and m^6^A modification in PE. Herein, we first demonstrated the severe ER stress state and upregulated m^6^A modification by increased METTL3 levels in preeclamptic placentas, and constructed an ER stress model with HTR-8/SVneo cells. Next, the effect of METTL3 on trophoblasts and its association with TMBIM6 were explored. Finally, we observed the therapeutic effect of METTL3 knockdown in rats with PE.

In this study, the expression levels of METTL3, GRP78, and CHOP were upregulated in preeclamptic placentas, which was consistent with previous studies (Wang et al. 2020, 2022; Marciniak et al. 2004; Hammadi et al. 2013). Moreover, the increase in METTL3 and m^6^A modification levels could contribute to trophoblast dysfunction, resulting in PE (Gu et al. 2021), and the ER stress state of the preeclamptic placentas was detrimental, leading to abnormal placentation during PE (Wang et al. 2022). Therefore, there were close links between ER stress and m^6^A modifications in PE.

Because placentas are composed mainly of trophoblasts and their dysfunction is the main cause of PE, we constructed an ER stress model in HTR-8/SVneo cells with TG to study their relationship. Moreover, the cell viability and expression levels of GRP78 and CHOP were measured at different times and concentrations to confirm their success. Consistent with the preeclamptic placentas, m^6^A modification and the expression levels of METTL3 were upregulated in HTR-8/SVneo cells under ER stress. However, METTL14, another methyltransferase for m^6^A modification, and FTO, a type of demethylase, showed no differences. Consequently, we used this cell model to further explore the association between ER stress and METTL3 expression in PE.

Subsequently, knockdown of METTL3 was performed with HTR-8/SVneo cells dysfunction reversed. Specifically, ER homeostasis was maintained during ER stress, and ROS production and apoptosis rates were reduced as their invasion ability increased. Therefore, METTL3 and mediated m^6^A modification were detrimental to trophoblast cells, as previously reported (Gu et al. 2021), whereas its downregulation had a protective effect. Next, we explored how METTL3 affected trophoblasts. Several studies have reported the protective effect of TMBIM6 against ER stress, and its mRNA was predicted to contain m^6^A sites and to be modified by METLL3 (Lebeaupin et al. 2020; Deng et al. 2021; Xuan et al. 2018). In addition, we demonstrated that TMBIM6 changed with METTL3 in both HTR-8/SVneo cells and placentas, and overexpression of TMBIM6 reversed HTR-8/SVneo cell dysfunction independently like the knockdown of METTL3. Therefore, there were close links between TMBIM6 and METTL3. Moreover, we further demonstrated that overexpression of TMBIM6 counteracted the detrimental effects of METTL3 on HTR-8/SVneo cells, indicating that TMBIM6 was a downstream regulator of METTL3.

Later, we explored a deeper mechanism about how TMBIM6 was regulated by METTL3. As previously reported, many “writers” such as METTL3 could transfer methyl groups to modified RNAs which were subsequently recognized by the “readers” to influence the cellular functions in m^6^A modification (Zhang et al. 2017, 2019). Among these “readers,” YTHDF2 was a regulator of mRNA circulation and could degrade the modified mRNAs by transporting them to the processing body in the cytoplasm (Zhong et al. 2019). This “reader” has been shown to accelerate the degradation of more than 3000 RNAs most of which were mRNAs (Wang et al. 2014). Here, we demonstrated that TMBIM6 could change with YTHDF2 in HTR-8/SVneo cells, and knockdown of YTHDF2 could improve the stability of TMBIM6 mRNA and upregulate the expression of TMBIM6 independently, similar to the knockdown of METTL3. Therefore, YTHDF2 played an important role in regulating modified TMBIM6 mRNA by METTL3. Moreover, we further demonstrated that YTHDF2 had no effect when METTL3 was downregulated. Therefore, we demonstrated a new mechanism that METTL3 could methylate TMBIM6 mRNA which was further recognized by YTHDF2 and downregulate the expression of TMBIM6, contributing to the dysfunction of trophoblast cells and development of PE.

Finally, based on this new mechanism, we knocked METTL3 down in vivo and demonstrated its therapeutic effects on PE. Specifically, we determined that the inhibition of METTL3 in preeclamptic rats could improve whole or local symptoms, such as high SBP, proteinuria, and renal and placental injury by decreasing m^6^A modification and upregulating TMBIM6 expression in the placenta. Moreover, inhibiting METTL3 could protect the fetus, improve the ER stress state, and reduce ROS production and apoptosis in preeclamptic placentas. This evidence supported targeting of METTL3 in PE treatment regimens.

Taken together, our study is the first to elucidate the relationship between the ER stress state in trophoblasts and METTL3 expression, and reveal a novel epigenetic mechanism in PE. Moreover, the therapeutic effects of targeting METTL3 were demonstrated, which represents a promising and attractive approach for treating PE. However, our study had some limitations that need to be considered. First, due to the difficulty in obtaining primary trophoblasts and their limited use, we replaced them with the HTR-8/SVneo cell line for this study. Therefore, there is a need to confirm our findings on primary trophoblasts in future studies. Second, our study could not reveal the mutations of METTL3 and TMBIM6 with PE development because of the ethical issue. Finally, we only demonstrated the therapeutic effects of METTL3 inhibition in preeclamptic rats. Therefore, further translational research on humans is essential.

## Conclusions

**Fig. 9:**
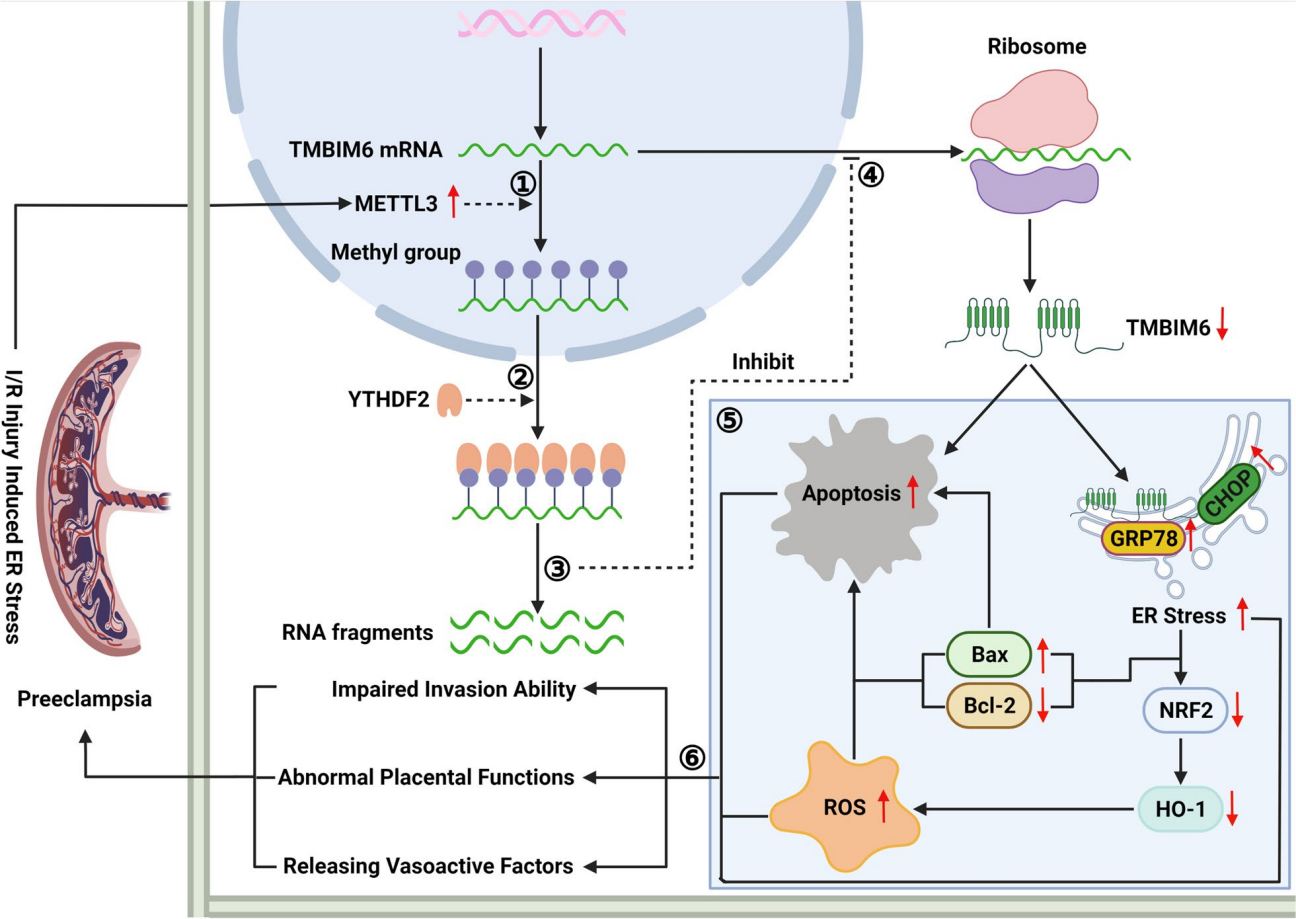
The schematic of molecular mechanism about how METTL3 affected TMBIM6 expression resulting in trophoblastic dysfunction and PE. ① When placentas got injured by many detrimental factors, such as I/R, the trophoblasts were under ER stress and METTL3 expression was upregulated increasing the m^6^A modification of TMBIM6 mRNA in trophoblasts. ② The methylated TMBIM6 mRNA was recognized by YTHDF2. ③ YTHDF2 decreased the stability of TMBIM6 mRNA and increased its degradation. ④ TMBIM6 expression was downregulated due to the decreased TMBIM6 mRNA and its translation. ⑤ Downregulation of TMBIM6 could not maintain the ER homeostasis, which aggravated ER stress. Moreover, the apoptosis and ROS production were increased when TMBIM6 was decreased. ⑥ The trophoblasts functions were impaired with the placental functions abnormal, which consequently contributed to PE

Herein, we uncovered a novel link between METTL3 and PE. Our results demonstrate that METTL3-driven m^6^A modification of TMBIM6 mRNA promotes the development of PE via YTHDF2 which recognizes methylated TMBIM6 mRNA and increases its degradation. Downregulation of TMBIM6 expression impairs the trophoblast function by aggravating ER stress and increasing ROS production and apoptosis (Fig. 9). Additionally, the therapeutic effects of METTL3 knockdown were observed in preeclamptic rats. The present findings reveal a novel epigenetic regulatory mechanism for PE. Therefore, targeting the METTL3/YTHDF2/TMBIM6 axis may be a promising therapeutic approach for PE.

## Supplementary Information

**Additional file 1: Table S1.** The sequences of PCR primers.

**Additional file 2: Table S2.** The sequences information of shRNA target gene.

**Additional file 3: Table S3.** The basic features of rats in four groups on GD9.

**Additional file 4: Figure S1.** The m^6^A sites of TMBIM6 predicted by RMBase v2.0.

**Additional file 5: Figure S2.** TMBIM6 was the potential target of METLL3 by M6A2Target.

## Data Availability

All data generated or analyzed during this study are included in this published article and its Additional files.

## Abbreviations

PE: Preeclampsia
ER: Endoplasmic reticulum
UPR: Unfolded protein response
ATF6: Activating transcription factor 6
ATF4: Activating transcription factor 4
CHOP: C/EBP homologous protein
PERK: PKR-like endoplasmic reticulum kinase
GRP78: Glucose-regulated protein 78
TMBIM6: Transmembrane Bax inhibitor Motif-containing 6
BI-1: Bax interactivator-1
ROS: Reactive oxygen species
METTL3: Methyltransferase-like family members 3
METTL14: Methyltransferase-like family members 14
WTAP: Wilms tumor 1-associated protein
FTO: Fat mass and obesity associated
YTHDF2: YTH m^6^A-binding protein 2
I/R: Ischemia–reperfusion
ACOG: American college of obstetricians and gynecologists
qRT-PCR: Quantitative real-time PCR
IHC: Immunohistochemistry
WB: Western blot
TG: Thapsigargin
MeRIP: Methylated RNA immunoprecipitation
CCK8: Cell counting kit-8
SPF: Specific-pathogen-free
L-NAME: L-nitro-arginine methylester
LV: Lentiviral vector
HE: Haematoxylin‐eosin
SBP: Systolic blood pressure
NRF2: Nuclear factor erythroid 2-related factor 2
HO-1: Heme oxygenase-1
Bcl-2: B cell lymphoma-2
Bax: Bcl-2-associated X protein
RNA-seq: RNA sequencing
MeRIP-Seq: Methylated RNA immunoprecipitation sequencing
SEM: Standard error of mean
