# Supplementary material for: Methyltransferase-like 3 aggravates endoplasmic reticulum stress in preeclampsia by targeting TMBIM6 in YTHDF2-dependent manner
Source: Mol Med. 2023 Feb 6;29:19. doi: 10.1186/s10020-023-00604-x (PMC9901113; doi:10.1186/s10020-023-00604-x)
Supplement: Supplementary file 1 — Additional file 1: Table S1. The sequences of PCR primers. [file 10020_2023_604_MOESM1_ESM.docx]

Table S1: The sequences of PCR primers.

| Primers | Source | Sequences | Applications |
| --- | --- | --- | --- |
| METTL3 | Human | Forward: 5’-TGCTTGGTTGGTGTCAAAGG-3’ | qRT-PCR |
|  |  | Reverse: 5’-AATCTTGCGAGTGCCAGGAG-3’ |  |
| TMBIM6 | Human | Forward: 5’-TTTGTATGTTTGTGGCGGCTG-3’ | qRT-PCR |
|  |  | Reverse: 5’-ATGGCTATGAGGTGTTGCCA-3’ |  |
| β-actin | Human | Forward: 5’-TCATATAACCCCGTCAACGC-3’ | qRT-PCR |
|  |  | Reverse: 5’-TCGGCCACATTGTGAACTTT-3’ |  |
| TMBIM6 | Human | Forward: 5’-TCATATAACCCCGTCAACGC-3’ | MeRIP-qPCR |
|  |  | Reverse: 5’-CAAATCCAGCAAGAAGTCCC-3’ |  |
| METTL3 | Rat | Forward: 5’- ATGTGCAGCCCAACTGGATT-3’ | qRT-PCR |
|  |  | Reverse: 5’-CTGTGCTTAAACCGGGCAAC-3’ |  |
| β-actin | Rat | Forward: 5’-TCATATAACCCCGTCAACGC-3’ | qRT-PCR |
|  |  | Reverse: 5’-TCGGCCACATTGTGAACTTT-3’ |  |
