# Supplementary material for: Methyltransferase-like 3 aggravates endoplasmic reticulum stress in preeclampsia by targeting TMBIM6 in YTHDF2-dependent manner
Source: Mol Med. 2023 Feb 6;29:19. doi: 10.1186/s10020-023-00604-x (PMC9901113; doi:10.1186/s10020-023-00604-x)
Supplement: Supplementary file 2 — Additional file 2: Table S2. The sequences information of shRNA target gene. [file 10020_2023_604_MOESM2_ESM.docx]

Table S2: The sequence information of target gene.

| sh-RNA | Specie | Target sequence information |
| --- | --- | --- |
| sh-METTL3-1 | Human | GCTGCACTTCAGACGAATTAT |
| sh-METTL3-2 | Human | GGTCTGAACTCTTCAGCATCG |
| sh-METTL3-3 | Human | GAAGACAAATCAACTGCAACG |
| sh-YTHDF2-1 | Human | GCCATGATTGATGGACAGTCA |
| sh-YTHDF2-2 | Human | ACACATTCGCCTAGAGAACAA |
| sh-YTHDF2-3 | Human | AGCAACCTAAACTGAAGACCA |
| sh-METTL3 | Rat | GCTGCACTTCAGACGGATTAT |
