# Supplementary material for: Methyltransferase-like 3 aggravates endoplasmic reticulum stress in preeclampsia by targeting TMBIM6 in YTHDF2-dependent manner
Source: Mol Med. 2023 Feb 6;29:19. doi: 10.1186/s10020-023-00604-x (PMC9901113; doi:10.1186/s10020-023-00604-x)
Supplement: Supplementary file 3 — Additional file 3: Table S3. The basic features of rats in four groups on GD9. [file 10020_2023_604_MOESM3_ESM.docx]

| Group | NC | PE | PE+LV-sh-Con | PE+LV-sh-METTL3 | p |
| --- | --- | --- | --- | --- | --- |
| The weight of rats(g) | 290.8± 1.21 | 289.4± 1.30 | 290.5± 2.60 | 288.6± 1.68 | ns |
| SBP (mmHg) | 97.17± 3.75 | 98.67± 2.40 | 99 ± 4.03 | 98.17± 4.175 | ns |
| Proteinuria(g/L) | 0.31± 0.018 | 0.29 ± 0.014 | 0.29 ± 0.018 | 0.28 ± 0.027 | ns |

Table S3. The basic features of rats on GD9 in four groups.
