# Supplementary figures and images for: Methyltransferase-like 3 aggravates endoplasmic reticulum stress in preeclampsia by targeting TMBIM6 in YTHDF2-dependent manner
Source: Mol Med. 2023 Feb 6;29:19. doi: 10.1186/s10020-023-00604-x (PMC9901113; doi:10.1186/s10020-023-00604-x)

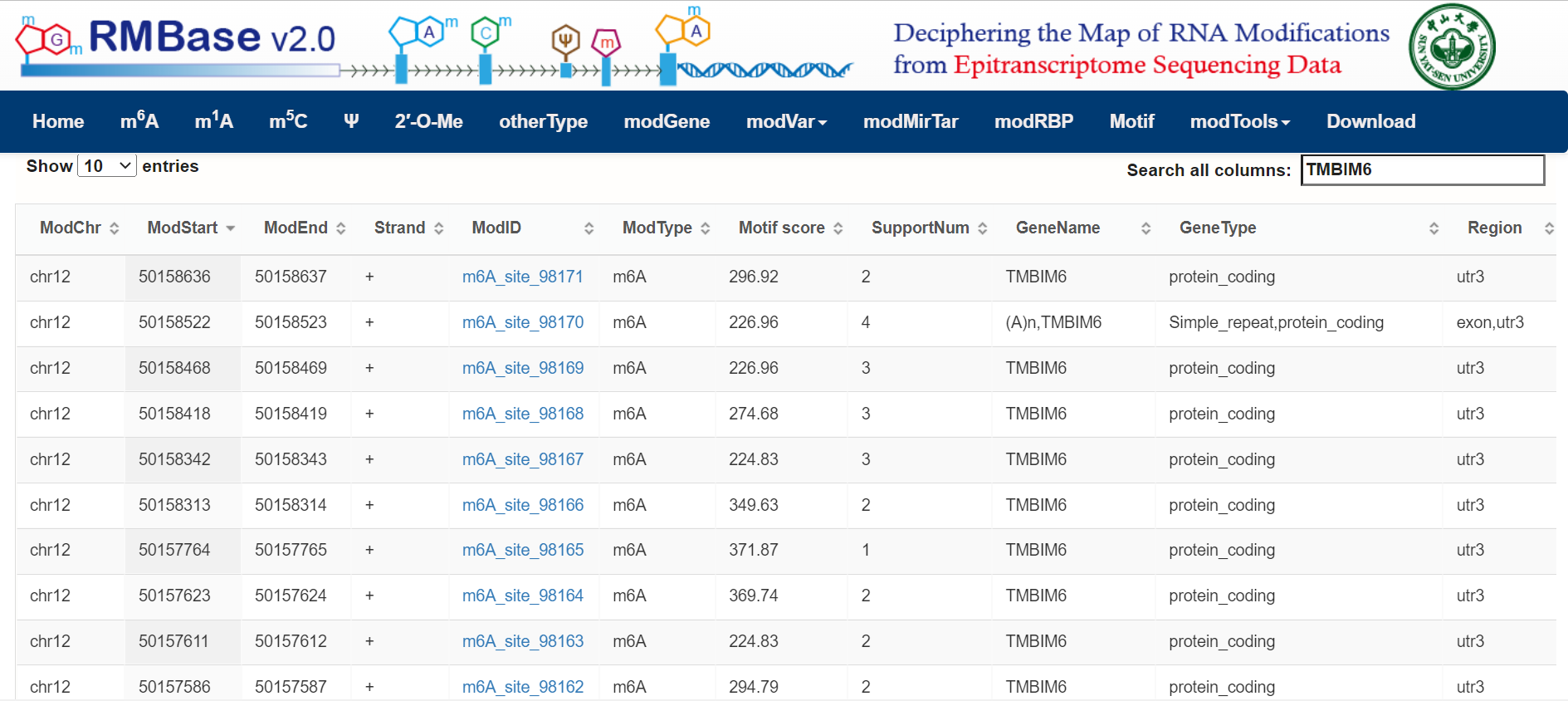

Supplement: Supplementary file 4 — Additional file 4: Figure S1. The m6A sites of TMBIM6 predicted by RMBase v2.0. [file 10020_2023_604_MOESM4_ESM.png]

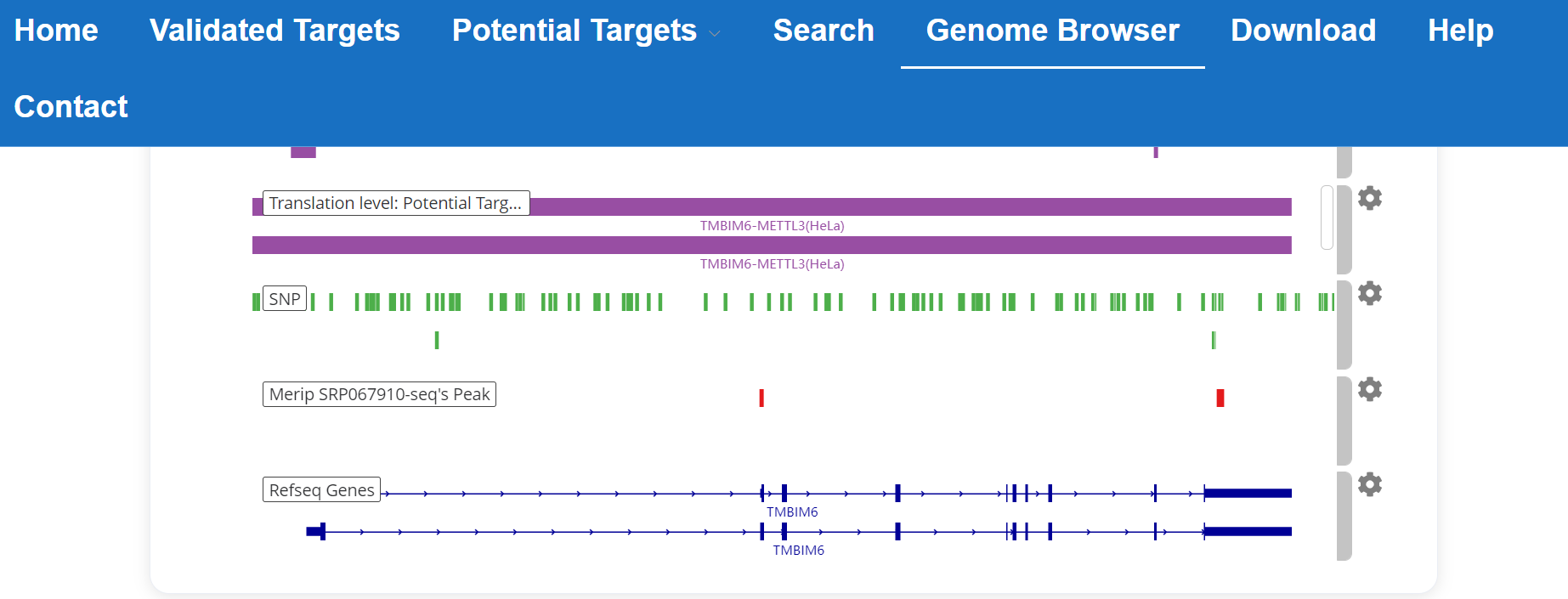

Supplement: Supplementary file 5 — Additional file 5: Figure S2. TMBIM6 was the potential target of METLL3 by M6A2Target. [file 10020_2023_604_MOESM5_ESM.png]
